# Transition to motherhood following the use of assisted reproductive technologies: Experiences of women in Ghana

**DOI:** 10.1371/journal.pone.0266721

**Published:** 2022-04-22

**Authors:** Kwadwo Asante-Afari, David Teye Doku, Eugene K. M. Darteh

**Affiliations:** 1 Health Promotion Division, Ghana Health Service, Accra, Ghana; 2 Department of Population and Health, University of Cape Coast, Cape Coast, Ghana; African Population and Health Research Center, KENYA

## Abstract

As a result of the significance of childbearing in the Ghanaian culture, couples would go to all lengths to have biological children. One of the means that has made it possible for childless couples to have children is through the use of various assisted reproductive technologies. Using a qualitative research design, the paper explores the experiences of 40 women who have delivered following the use of assisted reproductive technology in Ghana. A semi-structured interview guide was utilised to explore women’s experiences and results were analysed thematically. The study revealed that childless women faced hostile treatment but the birth of a child ceased the hostility, giving couples social recognition. The study also revealed that the transition to motherhood is characterised by excitement, high self-esteem, recognition and acceptance into spouses’ families. It was a source of anxiety for other women due to society’s perception of children born following the use of assisted reproductive technologies. However, women perceived that having a second or third child could change society’s perception about the use of assisted reproductive technologies to have children. Based on these assumptions, there is a need for public education to change the societal perception about women who utilise assisted reproductive technologies to meet their parenthood desires as well as children who are born following the use of assisted reproductive technologies.

## Introduction

Procreation is a vital event in the life of every individual, especially married couple. However, for several couples, procreation is not easily fulfilled due to infertility. Infertility occurs when a sexually active couple does not achieve pregnancy despite having unprotected sex and desiring to become pregnant for at least 12 months.

Globally, it is estimated that nearly 80 million adults of their reproductive ages are affected by infertility while about 15 percent of all marriages are affected by infertility [1]. Sub-Saharan Africa is one of the regions with the highest infertility prevalence [2], with differences in the prevalence levels by country. For example, Gambia has an estimated record of infertility rate of 9 percent, North-Western Ethiopia has 21.2 percent [3] and Nigeria has between 20 percent and 30 percent [4]. In Ghana, the infertility prevalence rate is 11.8 percent among women and 15 percent among men [5].

It has been argued that the effects of infertility are enormous [6–9]. For example, it is noted that the consequences of infertility may affect the health system and the delivery of its services such as the use of contraceptives [10]. At the individual level, childless women may face economic hardships, social segregation, violence, marital dissolutions, physical and emotional abuses [11–14]. Childless women may also be secluded from certain cultural practices and even at their death, special rituals may be performed to circumvent their infertility situation in the next world [15]. These practices depict that the entire society is against childlessness, hence their discontent and adverse attitudes towards people with infertility.

Based on these practices, women explore several avenues to have children, especially in their marriages. For example, some women allow co-wives in their marriages just to have children [16]. Divorce and remarriage to different spouses have also been observed as means of solving infertility challenges [17]. In some cultures, women who are not able to have offspring in their marital unions engage in extramarital unions to have children for their husbands [18] whilst others depend on the adoption of children as an alternative way of coping with childlessness [19]. The use of herbal and/or complementary and alternative medicines (CAM) to treat infertility or enhance fertility is also practised [20]. Studies in Cameroon [21] and Nigeria [22] have all documented the use of herbal and/or CAM to treat infertility and/or enhance fertility.

In recent times, many individuals who are faced with infertility use orthodox medical therapies, including the various Assisted Reproductive Technologies (ART). Assisted Reproductive Technology is defined as ‘all interventions that include the *in vitro* handling of both human oocytes and sperm or of embryos for reproduction’ [23]. By 2010, 55 percent of countries in the world were offering In-Vitro Fertilisation (IVF) services [24]. It is estimated that about 4000–4500 IVF centres existed worldwide as of 2015. Japan and India had between 606 and 618 and about 500 centres respectively. In the USA, it is estimated that about 450–480 fertility treatment clinics exist. Italy has recorded about 360 facilities while Spain has between 177 and 203. Korea and Germany have 142 and between 120 and 121 IVF facilities respectively [25].

In Africa, due to a dearth of information, it is challenging to estimate the total number of IVF centres currently operating. However, it is believed that 21 out of the 54 countries in Africa have at least a registered IVF unit. Countries with the highest number of IVF centres in Africa include Egypt (52), South Africa (37), Nigeria (12), Ghana (8), Uganda (4), and Kenya (3) while the rest of the countries have at least one each [26].

Due to the recent increases in the numbers of fertility treatment hospitals in the world, pregnancies and deliveries as a result of the use of ART continue to increase. For example, it is estimated that the use of ART accounts for 1.7 to 4 percent of all pregnancies whereas the collective summation of births as a result of the use of ART is about 5 million globally [27]. In Africa, there is a paucity of information about the total number of children born through the use of ART but it is believed that the use of ART is largely accepted as a means of overcoming infertility [28].

In Ghana, there is limited information, especially on the number of pregnancies and deliveries following the use of ART. However, there are indications that ART usage dates back to the mid-1990s. It was not popular until public discourses and research interest began increasing [29, 30]. Despite the few scientific studies in this relatively new area, a study on the experiences of women who have given birth following the use of ART in Ghana is still not available. The present study attempts to contribute to the literature by exploring the experiences of women who have delivered with ART.

## Theoretical review

The model of help-seeking for infertility was adopted as the framework for the study [31]. According to [31], the desire to seek medical treatment for infertility is directly influenced by symptom salience, life-course variables, individual and social cues, as well as predisposing and enabling factors. Related to this paper, the model considers the problem and severity of infertility symptoms as key factors that influence the pursuance of medical treatment, including the use of ART. One of the prominent challenges experienced by infertile women is stigma and adverse treatment by society. Women also suffer physical abuses and violence [13] while others are secluded from certain cultural practices even at death [15]. Although symptoms recognition in infertility may be difficult to identify as compared to other forms of sicknesses, it is assumed that women with infertility go through emotional and psychological challenges. These challenges may have detrimental effects on their health and may influence the decision to seek infertility treatment.

Additionally, the influence of life course cues was highlighted by the model as a factor that may affect seeking treatment for infertility. For example, a young unmarried woman may not envisage infertility even though she may be indulging in unprotected sexual intercourse. If there is no pregnancy, the tendency to seek treatment may be lower compared to a middle-aged married woman who may be having unprotected sex without conception. Parity was also considered under life course cues. Here, it is explained that the number of children one has is likely to influence the decision to seek medical treatment for the delay in pregnancy and childbirth. Women who experience primary infertility are likely to seek early help compared to others who have ever given birth or are having at least a child. This is because an additional birth decreases the perception of infertility. An individual’s social network plays a key role in deciding for a social member who is experiencing infertility to seek treatment. Network ties promote various resources, including emotional, informational and instrumental support. In the case of infertility, the partner’s desires may likely be paramount to influence the decision to seek help; friends and society may also encourage medical help-seeking.

Predisposing conditions such as education, health locus of control, general health and attitudes toward treatment are likely to influence the decision to seek infertility treatment. An individual’s level of education may have a direct influence on information search on the causes of infertility and where to seek treatment [32]. Medical locus of control, which signifies the likelihood for individuals to perceive their health to be either controlled by their influence (internal locus) or external forces such as medical practitioners, is likely to impact help-seeking behaviour. Knowledge, awareness and acceptability of the available treatment for infertility are also key. According to [33], when people are aware of treatment availability, cost and the possible health implications, they are likely to seek treatment for their infertility conditions. Acceptability of ART treatment, which relates to an individual’s willingness, is another factor that influences decision-making. This is mainly influenced by socio-economic factors such as religion, culture and social distance.

Enabling conditions such as income, health insurance and location influence the decision to utilise treatment. To a greater extent, income and wealth promote better health because the affluent could afford resources that could prevent sickness and also seek treatment anytime they are affected. Another key determinant of access to health care utilisation is health insurance. In most cases, conditions that are fully or partially covered by health insurance are likely to be reported at health facilities for care. In the case of Ghana, issues of infertility treatment and the use of ART are not covered by the National Health Insurance Scheme (NHIS). As a result, infertility treatment in Ghana is paid for by individuals who are affected. Taking these variables into consideration, it appears that culture, economics, access, perceptions, knowledge and literacy, belief in efficacy, age, gender, social roles and even the health system are among the factors that influence the decision to seek treatment.

## Material and methods

The study was conducted in five fertility hospitals in Ghana. Three of these hospitals (Lister Hospital and Fertility Centre, Tema Women’s Hospital, and Finney Hospital and Fertility Centre) are located in Greater Accra, and the other two facilities (Ruma Fertility and Specialist Hospital, and Trustcare Specialist Hospital) are located in Kumasi, the second-largest city in Ghana. These facilities are among the first to be established in the country and have provided services for more than ten years.

A qualitative research design using semi-structured interviews was adopted to explore the experiences of women who had delivered through the use of ART. The interviews focused on feelings and thoughts about childlessness, experiences about pregnancy, mode of delivery and motherhood. A total of 40 women (8 each from the five hospitals) who had successfully given birth through ART were purposely selected for the study. In recruiting respondents, the records of patients’ consultations and interactions with the medical team were verified in the selected hospitals. Inclusion criteria for participants embraced only women with medical records, were mentally sound, had undergone ART treatment with the selected facilities and were willing to participate in the study. Meetings were scheduled with participants who agreed to be part of the study. Information about the study, use of the data being collected and an estimated time frame of the interview was made known to the participants.

An in-depth interview guide was used to solicit information about the experiences of respondents. Interviews were done in the various health facilities. The interview guide was categorised into themes of inquiry, which included socio-demographic profile, feelings and thoughts about their childlessness period, pregnancy and method of delivery and experiences about motherhood. Written consent was sought from participants before the start of the interviews and for audio documentation of the interviews. Despite this process, two respondents initially were hesitant to be part of the study for fear of disclosure of their identity. However, after the explanation of the purpose of the study, they accepted to be part and signed the written informed consent form.

Participants were interviewed in the English language and Twi (a Ghanaian dialect) in a free atmosphere without social desirability bias. Confidentiality of respondents and their information was maintained throughout the study. A total of 40 participants were interviewed until data saturation was reached. Interviews conducted in the local dialect were accurately translated using the back-translation method and transcribed into the English language. Gaps identified during this process were filled by referring to field notebooks and interview tapes whenever there was a need. Peer debriefing, member check and stepwise replication approaches were also used to ascertain auditory files and transcribed interviews. NVIVO software version 12 was employed to run the codes and categorise themes and sub-themes that emerged from the interviews. Data were analysed thematically using the six phased approach to thematic analysis [34].

### Ethical considerations

Ethical approval was obtained from the University of Cape Coast Institutional Review Board with approval number UCC/IRB/A/2016/54 and the Ghana Health Service Ethics Review Committee with approval number GHS-ERC: 02/10/2016.

## Results

### Background characteristics of respondents

Twenty out of the total respondents (40) were aged 40–49 years, 13 were within the ages of 30–39 years while seven respondents were 50+ years. Twenty-two (22) respondents had secondary education, 13 had completed tertiary and five had completed either Junior High School or Middle School. The majority of respondents (38) were married while two were widowed. Also, 29 of the respondents were Christians, nine were Muslims and two did not belong to any religion. Table 1 presents details of the background characteristics of respondents.

**Table 1:** Background characteristics of respondents.

Demographic Characteristics	Number
Age group	
30–39	13
40–49	20
50+	7
**Education**	
JHS/Middle	5
Secondary	22
Tertiary	13
**Marital status**	
Married	38
Widowed	2
**Religion**	
Christian	29
Islamic	9
No religion	2

### Relationship with husband before childbirth

Due to the premium put on children in Ghanaian society, marriages without childbirth are regarded as a serious challenge that affects the couple, their families and the society at large. The relationship between a couple before childbirth ranged from spousal abuses and, in some cases, support from husbands. A woman who experienced a supportive relationship with the spouse had this to say:

*‘My husband was very supportive. He took me to wherever we heard we could be assisted to get a baby even till we heard about IVF’*

(Respondent 9)

A 40-year-old respondent whose husband stood behind her when her in-laws wanted to throw her out of her marriage shared her experience:

*‘My husband was a pillar behind me when his family decided to throw me out of the marriage. He accepted the blame that our inability to have a child was his fault just to save the marriage.’*

(Respondent 23)

A 40-year-old respondent whose husband regarded her inability to conceive as a punishment from God shared her experience:

*‘My husband quarrelled with me all the time and told me that my situation was a punishment from God because I stopped from the seminary to get married to him. This same man who persuaded me to leave the seminary now turns against me because of my inability to have a child’*

(Respondent 4)

Another respondent whose husband always picked up quarrels with her because she was not able to conceive had this to say:

*‘I have never known peace for the past 9 years without a child. He threatens me with divorce anytime there is a misunderstanding but I knew the problem came from him; he will simply not accept it’*

(Respondent 17)

### Relationship with in-laws before childbirth

In the Ghanaian culture, mothers-in-law and other family members significantly influence the decision to marry and procreation-related issues. A respondent whose in-laws pressurised and married another woman for their son to have a child shared her experience:

*‘My husband is a Traditional leader and customs demand that he gets a child preferably a son to succeed him. As a result, his family married another woman for my husband just to have a child so that they could preserve the family’s lineage. God is so good, I have given birth to a male and my rival gave birth to a female’*.

(Respondent 1)

### Method of child delivery

All respondents who conceived through the use of ART gave birth through caesarean section. Various reasons have been cited to explain why women went through caesarean sections. For example, a 45-year-old mother narrated that:

*‘The pregnancy was so precious to me that I did not want to lose it.’*

(Respondent 6)

Another respondent who could not deliver spontaneously due to her advanced age had this to say:

*‘I am old and I am afraid that I may find it very difficult to deliver spontaneously.’*

(Respondent 33)

A 49-year old mother who, for medical reasons, delivered through the caesarean section indicated that:

*’For medical reasons, the doctor advised me to deliver through a caesarean section’*.

(Respondent 10)

### Motherhood

In Ghanaian society, motherhood is celebrated. As a result, women employ various means to achieve motherhood because of the importance attached to children. Mothers expressed their feelings about motherhood. A 47-year-old mother had this to say:

*‘Being a mother is the sweetest. Recently, I felt sick and surprisingly my little girl pulled the rosary and prayed for me. At this point, I realised that someone cares about me’*.

(Respondent 18)

A 45-year old mother recounted the joy she had when her child first called her ‘mummy’:

*The very first day my child called me ’mummy’, I felt so happy and tears of joy flowed just like that. I said ’God thank you I have been called mummy at last’. I will be called mummy for the rest of my life*.

(Respondent 6)

Other women mentioned that motherhood has helped them to gain recognition. A 40-year old mother stated:

*When people see and greet me I feel so good and reply to them easily and with all truthfulness. There were situations where I intentionally avoided greeting people because they may ask about the health of my children which I did not have by then*.

(Respondent 27)

### Relationship with husband after childbirth

All the women confirmed that the attitudes of their husbands changed after childbirth. Husbands provided for their needs and supported them in caring for their children. A 41-year old mother confirmed that:

*The clock has turned; my husband is always with me. I never knew childbirth could change everything in the house*.

(Respondent 37)

Another mother whose husband started playing the role of a father had this to say:

*‘My husband has stopped seeing other women and the care for the child and I is great’*

(Respondent 30)

### Integration into the family after childbirth

After childbirth, the entire community and family members come together to celebrate with the couple. A mother narrated how she gained social recognition after childbirth:

*‘I feel very good and proud in my community because I am counted among mothers. What is better than this?’*

(Respondent 10)

A respondent whose relationship with her in-law had become better shared her experience:

*’My in-laws who were against me have apologised to me. They say it was a way to force me to get a child for their son.’*

(Respondent 36).

Other women had challenges with their families even after successful childbirth following the use of ART. A respondent narrated how her relationship with her families and the people in her communities had worsened after childbirth:

*‘My family and neighbours accused me of buying babies. This forced me to relocate.’*

(Respondent 2)

A respondent who lost her husband before she delivered shared her experience:

*Could you believe that when I delivered after the death of my husband, my family and in-laws accused me of using my husband for the birth ritual?’*

(Respondent 25)

### Intention to give birth again

Whilst women who gave birth for the first time indicated that they would like to give birth again, others who had multiple births were content. A mother reported that:

*‘I would like to have a second child and this will prove to people that I am not barren as they perceived me’*

(Respondent 11)

Another mother who needed a second child had this to say:

*‘I will be more than grateful to God to have another child; I don’t want my child to be lonely. I want at least two children so that they can care for me when I am old.’*

(Respondent 20)

Another mother who had decided not to have a second child due to her age indicated that:

*‘I am now 50 years old; I am not as strong as before and so I have to end it. I am okay with twins’*

(Respondent 39)

A woman who had delivered triplets had this to say:

*’I don’t have any intention to have another child. Triplets are okay for me. Instead, God should give it to other women who are struggling to have children.’*

(Respondent 13)

## Discussion

Results from the study indicate that husbands and their families, especially mothers-in-law, and even the entire society are hostile and stigmatised women who are childless in their marriages. This could be attributed to the notion that childless women had led promiscuous lives, committed abortions or have been cursed whilst men are always considered fertile [35]. Other studies have confirmed that childless women often show signs of anxiety, stress, depression, social labelling and loss of social recognition [7–9], which often results in self, gender and social discrepancies. This attitude may be due to the belief that the purpose of marriage was not complete until couples begin to procreate [36]. Again, the patrilineal family system of inheritance, which is common in Ghanaian society, could be a cause of the unfriendly treatment meted out to women in marriages, especially by the spouse’s family.

The study also found that the birth of a child and transition to motherhood restored the dignity of the women in the matrimonial homes and society. This means that childbirth is crucial, especially in the traditional society, and may have a significant impact on the mental health and well-being of women, especially in marriages. This suggests that childbirth consolidates and stabilises women’s status within the marital home, family and community settings. Childbirth as a result of a successful ART treatment also reduced women’s anxiety, depression and stress. Accordingly, the sense of happiness experienced by women who successfully have children following the use of ART tends to outweigh any other challenges in life [37]. As a result, these women may often become overprotective and overly concerned, especially about the health and well-being of their children.

The study further revealed that women who got pregnant following the use of ART delivered through caesarean sections (CS). Caesarean deliveries were either recommended by obstetricians or opted for by the women themselves. A previous study identified that both women and their obstetricians are usually hesitant to go through spontaneous deliveries after ART treatment due to the perceived risks [38]. In the present study, it was revealed that the majority of women opted for CS due to their advanced ages (40–50 years). Other studies have pointed out that planned CS rates were nearly threefold in women of advanced age who went through ART in Taiwan [39]. At the same time, one out of every five obstetricians confirmed carrying out CS at the wish of older women who conceive after successful ART treatment [40, 41].

Other women perceived their pregnancy to be precious to them considering the cost in terms of time, funds, emotions and the treatment procedures they had gone through. Such women avoided going through vaginal deliveries which were perceived to be painful and, therefore, opted for CS. It has been reported that women opt for CS to avoid labour pains [42]. Caesarean deliveries were also recommended for women by the medical team because it was thought to be safer, especially for women who had multiple pregnancies [43].

The study also disclosed that the birth of a child and the transition to motherhood generated a sense of joy, completeness and family re-interconnections. A similar study in Ghana found that maternal transition served as the source of fulfilment of personal joy, marital satisfaction and the way a wife becomes accepted into the husband’s family [44]. Despite the characteristics that accompany this feat, a maternal transition could be described as a critical stage in the lives of women due to the postpartum responsibilities.

The study further showed that while the delivery of a child through the use of ART resulted in excitement and integration of some mothers into their spouses’ families and the bigger society, it was a source of anxiety on the psychosocial health and emotions of other women. This is mainly due to society’s perception about children born following the use of ART. Some perceive children born through the use of ART to have birth and psychological development defects. Others questioned the appropriateness of the technology and possible health risks associated with the procedure due to human interference with the natural procedure of conception and delivery. Others also perceived that children born following the use of ART may have low IQ which may affect the quality of life in adulthood. As a result, some women conceal their fertility challenges, the use of ART services, pregnancies and deliveries. Concealing infertility and treatment with ART as a result of societal perceptions has been reported among women in Iran [45].

The results of the study also showed that women who gave birth through the use of ART for the first time had the intention to give birth again, probably to fulfil the fertility replacement principle. Women also considered the birth of a second or third child as a way to prove to friends, family and society that they were not infertile as they were perceived. For other women, it was necessary to give birth to two or more children so that their homes will become lively while others maintained that two or more children could better cater for them in their old ages. This finding corroborates the old-age security proposition which maintains that in the absence of capital markets, children serve as an asset that allows parents to transfer income to old age. From this perspective, parents continue to give birth to safeguard economic support in their old age. Despite these assertions, other women who had delivered multiple children did not have any intention to give birth again because they had got what their hearts desired or as a result of their advanced ages.

In considering the findings of the study, some limitations must be addressed. First, only women were considered for the study although infertility is medically addressed as a couple-related issue. Additionally, only women with successful birth outcomes were included in the study thus limiting the generalisability of findings. Despite these limitations, the study has contributed to the filling of the literature gap on the use of ART in Ghana where this technology is relatively new.

## Conclusion and recommendations

The overall findings from the study showed that women who are childless faced disapproval from families, friends and society. However, the birth of a child brought to an end the hostile treatment meted out to childless women. Motherhood was considered as self-fulfilment, ensuring marital security, the continuation of family lineage and commanding respect from society. This means that the treatments given out to women who are childless in their marriages could be lessened if society is made to appreciate that the use of ART could help them have children. The study, therefore, suggests the need for public education and social support interventions for families and women who go through ART treatments. It will also be prudent to provide proper counselling services at the various stages of fertility treatments and the transition period to deal with the anxiety of women.

## Supporting information

S1 File (DOCX)
