# Supplementary material for: Transition to motherhood following the use of assisted reproductive technologies: Experiences of women in Ghana
Source: PLoS One. 2022 Apr 22;17(4):e0266721. doi: 10.1371/journal.pone.0266721 (PMC9032403; doi:10.1371/journal.pone.0266721)
Supplement: S1 File — (DOCX) [file pone.0266721.s001.docx]

**S1 File**

**In-depth interview schedule for women who have delivered through the use of Assisted Reproductive Technologies**

1. **Socio-demographic profile**

Age

Sex

Education

Marital status

Duration of marriage before delivery

Religion

1. How long have you tried to have a child without success before the decision to go for ART?
2. What was your relationship with your husband, in-laws, friends and family when you had no child? Probe further on the answer/answers given
3. What has changed with your husband, in-laws, family and friends since you had a child?
4. What about your relationship with other members of your community then and now?
5. What kind of support did you receive from being a member of a group (*if any*) when you had no child? Probe to find out the benefits she received from
   1. Religious groups
   2. Other social groups
6. How does it feel to be a mother?
7. Tell me your experiences about motherhood. Probe on the experiences about routine activities, new roles and responsibilities
8. How was your baby born? Probe to find out whether the child was delivered spontaneously or through a caesarean session and why?
9. I will want to know if you have had a child or you intend to have a child/children after your first child.
10. What will be your reasons for intending to/not to have more children?
11. How have you integrated into your community especially with your new status as a mother? (probe for specific family members and other people)
12. What will be your major form of assistance to people who do not have children in your community?
13. Please share with me any other issues on your status as a mother that you want me to know.
